# Supplementary material for: Physiological effects of heat stress on Hawaiian picture-wing Drosophila: genome-wide expression patterns and stress-related traits
Source: Conserv Physiol. 2015 Feb 18;3(1):cou062. doi: 10.1093/conphys/cou062 (PMC4778489; doi:10.1093/conphys/cou062)
Supplement: Supplementary Data [file cou062.zip]

Conservation Physiology
Volume 3 • 2015 10.1093/conphys/cou062
SEB Society for Experimental Biology
Research article

Physiological effects of heat stress on Hawaiian picture-wing *Drosophila*: genome-wide expression patterns and stress-related traits

Karen L. Uy[1,*], R. LeDuc[3], C. Ganote[3], Donald K. Price[1,2]

[1]Tropical Conservation Biology and Environmental Science Graduate Program, University of Hawaii at Hilo, Hilo, HI 96720, USA
[2]Department of Biology, University of Hawaii at Hilo, Hilo, HI 96720, USA
[3]National Center for Genome Analysis Support, Indiana University, Bloomington, IN 47405, USA

***Corresponding author:** Tropical Conservation Biology and Environmental Science Graduate Program, University of Hawaii at Hilo, Hilo, HI 96720, USA. Email: karenlizeluy@gmail.com

Climate change is compounding the threats to the future of biodiversity, already impacted by habitat loss, invasive species and diseases. In the Hawaiian Islands, many of the endemic species have narrow habitat ranges that make them especially vulnerable to climate change. The Hawaiian *Drosophila*, a remarkably diverse group of species with 11 listed as federally endangered, are thought to be sensitive to temperature changes. To examine the species differences in sensitivity of Hawaiian picture-wing *Drosophila* to temperature changes, wild populations of *Drosophila sproati*, a relatively common species, and *Drosophila silvestris*, a rare species, were collected from two locations on Hawaii Island and bred in common laboratory conditions. Adult flies were exposed to hot and cold temperatures and compared with adult flies at control temperatures. *Drosophila silvestris* adults were less tolerant to heat stress than *D. sproati* for both survival and sperm mobility. In contrast, *D. silvestris* adults were more tolerant to cold stress than *D. sproati* for adult survival. The expression of 4950 Gene Ontology annotated gene transcripts was also analysed in high-temperature-treated and control males to identify candidate genes related to heat tolerance. There were more than twice as many transcripts differentially expressed after high temperature treatment for *D. silvestris* (246 transcripts) as for *D. sproati* (106 transcripts), with 13 Gene Ontology terms enriched between temperatures for *D. silvestris* and merely three in *D. sproati*. The combined results are consistent with *D. sproati* occurring more widely today as well as occurring at lower elevations than *D. silvestris* and with a genetically based temperature response, which is more severe in *D. silvestris* at high temperatures than that in *D. sproati*. These experiments demonstrate the potential for different capacities of species to adapt to future climate change conditions as well as providing an explanation for historical changes in the distribution of species.

Key words: gene expression, Hawaiian *Drosophila*, local adaptation, microarray, sperm mobility, temperature tolerance

Editor: Steven Cooke

Received 24 July 2014; Revised 14 December 2014; accepted 19 December 2014

Cite as: Uy KL, LeDuc R, Ganote C, Price DK (2015) Physiological effects of heat stress on Hawaiian picture-wing *Drosophila*: genome-wide expression patterns and stress-related traits. *Conserv Physiol* 3(1): cou062; doi:10.1093/conphys/cou062.

Introduction

The impact of global warming is likely to have important consequences for tropical species, especially for tropical insects (Bale *et al.*, 2002; Parmesan, 2006; Tewksbury *et al.*, 2008; Rodríguez-Trelles *et al.*, 2013). Global studies of tropical and temperate ectotherms have found that climate change may be more deleterious for some tropical species because of their

© The Author 2015. Published by Oxford University Press and the Society for Experimental Biology.
This is an Open Access article distributed under the terms of the Creative Commons Attribution License (http://creativecommons.org/licenses/by/4.0/), which permits unrestricted reuse, distribution, and reproduction in any medium, provided the original work is properly cited.

typically narrower critical maximal and minimal temperature tolerances, especially for those that live in less seasonally variable environments (Parsons, 1989; Deutsch *et al.*, 2008). Climate change may have additional impacts by altering the critical environmental cues required by insects throughout their life cycle during key developmental stages and to maintain synchrony with their host plants (Uvarov, 1931; Bale *et al.*, 2002; Robinet and Roques, 2010). Thus, it is important to determine how tropical insects are affected by temperature in order to understand better how climate change may alter tropical insects and the ecological communities in which they live.

Hawaii is home to a diverse community of endemic and indigenous species that have been negatively impacted by habitat changes, recently introduced species and the disruption of ecological communities over the past centuries. This has resulted in numerous species extinctions and a high number of threatened and endangered species (Vitousek *et al.*, 1987; Woodworth *et al.*, 2005; Price and Muir, 2008; Asner, 2013). Climate change has further potential to impact the endemic flora and fauna in Hawaii negatively. In the past 30 years, the air temperature in Hawaii has been increasing steadily (Giambelluca *et al.*, 2008), with projected greater warming at high elevations (Lauer *et al.* 2013). In 1960, the atmospheric CO_2 concentration measured at the Mauna Loa Observatory in Hawaii was below 320 ppm. Since then, it has increased steadily, reaching 401 ppm in 2014 (http://www.esrl.noaa.gov/gmd/ccgg/trends/). Climate models yield estimates that CO_2 concentrations will continue to increase at the rate of ~1.9 ppmv/year (IPCC, 2007). Environmental changes in Hawaii are already affecting some groups of organisms. For example, most Hawaiian honeycreepers have been forced to move to higher elevations to escape the advance of mosquitos carrying avian malaria (Jokiel and Brown, 2004; Atkinson and LaPointe, 2009). The Haleakala silverswords (*Argyroxiphium sandwicence*) have also recently been shown to experience climate-associated mortality in alpine habitat zones (Krushelnycky *et al.*, 2013). In addition, warming temperatures threaten to disrupt the predator–prey relationship of Hawaiian damselflies (*Megalagrion calliphya*) and invasive southern house mosquitos (*Culex quinquefasciatus*) in Hawaiian anchialine pools, which favours the mosquitos by releasing aquatic larvae from predation pressure by predatory nymph damselflies (Hobbelen *et al.*, 2013). However, the effect of climate change on many organisms, such as endemic Hawaiian *Drosophila*, are largely uncertain due to incomplete knowledge of temperature tolerances and future changes in the many interacting components of ecosystems.

Hawaiian *Drosophila* are a group of endemic insects that have experienced significant declines in overall densities and number of species in many habitats and currently, one threatened and 11 endangered species are in the US Federal Endangered species list (United States Fish and Wildlife Service, 2013 Endangered Species Database). In the past 30 years, a decline in Hawaiian picture-wing *Drosophila* species diversity was documented in the Olaa wet rainforest on Hawaii Island. Four of 14 species of picture-wing *Drosophila* that were present in the 1971–1972 survey were not observed in the 1992–1993 survey, including *Drosophila heteroneura*, a federally listed endangered species, and *Drosophila silvestris*. Conversely, *Drosophila sproati* was more frequently observed in the 1992–1993 survey but less observed in the earlier surveys, 1971–1972 (Foote and Carson, 1995). A similar situation can also be observed in the Saddle Road area in the east side of the Hawaii Island, where *D. sproati* is substantially more common than *D. silvestris* (K. L. Uy and D. K. Price, personal observation). Competition between cosmopolitan *Drosophila* species and endemic *Drosophila* species, predation from introduced western yellow jackets (*Vespula pennsylvanica*) and host plant decline due to feral ungulates are some factors that may have caused the decline in species diversity (Foote and Carson, 1995). In addition, the warming seen in Hawaii over the past 30 years (Giambelluca *et al.*, 2008) could contribute, in combination with the above factors, to the observed shift in species composition and decline of some Hawaiian *Drosophila* species.

It is well known that many *Drosophila* are affected by changes in temperature. The genus is known to develop adaptive shifts in distribution that lead to species differentiation from exposure to temperature extremes (Parsons, 1989; Hoffmann *et al.*, 2003). An extensive laboratory study on *Drosophila buzatii* and *Drosophila simulans* found that heat-related traits are under strong selection for local adaptation (Sarup *et al.*, 2009). Other *Drosophila* species are sensitive to small changes in temperature. For example, tropical populations of *Drosophila melanogaster* and *D. simulans* are more susceptible to heat and cold stress than their temperate counterparts. This is likely to be due to adaptation to more constant temperatures in tropical environments (Hoffmann, 2010). *Drosophila pseudoobscura* has also been shown to exhibit chromosomal inversion polymorphism patterns that reflect warm-climate gene arrangements that are increasing in frequency in European as well as American populations associated with the rise in temperatures due to global warming (Rezende *et al.*, 2010) and in a heat wave in 2011 (Rodríguez-Trelles *et al.*, 2013).

The Hawaiian montane cloud forests, home to many picture-wing *Drosophila*, including *D. sproati* and *D. silvestris*, are considered to be sensitive to environmental changes. Small changes in climactic patterns dramatically influence temperature and humidity within the forest (Loope and Giambelluca, 1998). However, it is unclear how Hawaiian *Drosophila* are reacting to the changes in temperature and humidity. One species, *D. silvestris*, has been shown to have chromosome inversion pattern changes associated with elevation changes that may reflect differential adaptation to temperature (Craddock and Carson, 1989). In *D. sproati*, a more common Hawaiian *Drosophila*, there is preliminary evidence of differential adaptation to clinal variation between populations found at different elevations (Eldon, 2010). This suggests that climactic variables associated with elevation may play a role in the ecology and evolution of the Hawaiian *Drosophila*. Therefore, it is important to determine whether the warmer climate expe-

rienced over the past 30 years in Hawaii, and which is likely to continue to change in the near future, with greater warming at high elevations, could alter the species diversity of Hawaiian *Drosophila*. In this study, we tested the hypothesis that *D. sproati*, a common and currently more widespread species, is more heat tolerant than *D. silvestris*, which has been declining in abundance and is currently a rare species. To test this hypothesis, we examined several physiological traits and overall gene expression patterns to identify key traits and genes that may be indicators of temperature tolerance in the two species.

Materials and methods

Collection of Hawaiian picture-wing *Drosophila*

Drosphila silvestris and *D. sproati* were collected from natural populations on the eastern (Saddle Road; SR) and western (South Kona Forest Reserve; SKFR) sides of Hawaii Island from April to September 2010. Flies were captured on fermented banana and mushroom-baited sponges that were hung from trees or tree ferns 1–2 m off the ground (Carson, 1982). All flies were captured alive using aspirators and transported in glass vials to the University of Hawaii at Hilo, where they were placed in a common laboratory environment and allowed to breed. Geographical co-ordinates were determined with a global positioning system as follows: from the eastern side of Hawaii Island, Saddle Road (N19 40.202, W155 20.142); and from the western side, South Kona Forest Reserve (N19 18.447, W155 49.098) in wet forest habitat at 1400–1500 m elevations. The SR collection site is located in an area with average annual rainfall of 2683.5 mm and average temperature of 12.5°C (range 3.6–25.8°C in September and 0.0–22.0°C in February). The SKFR area receives 798.8 mm average annual rainfall and has an average temperature of 14.4°C (range 5.4–25.8°C in August and 0.0–21.8°C in February; Giambelluca *et al*., 1986, 2014; http://climate.geography.hawaii.edu/interactivemap.html). Both locations are populated with large Ohia trees (*Metrosideros polymorpha*), providing shade and cooler atmosphere, as well as large tree ferns (*Cibotium* spp.) covering most of the understory. The SKFR location is more open and less wet than the SR location. Both east and west collection sites harbour Olapa trees (*Cheirodendron trigynum*), which are known to be the host plant of both *D. silvestris* and *D. sproati*. Breeding females deposit eggs in decaying Olapa bark (Magnacca *et al*., 2008). The SKFR location, besides the abundance of Olapa trees, also harbours semi-woody *Clermontia clermontioides*, another known host plant of *D. silvestris*. *Drosophila sproati* are known to be monophagous to the family Araliaceae, while *D. silvestris* have been found to breed in both Araliaceae and Campanulaceae (Magnacca *et al*., 2008). Both sites represent different populations of both species; therefore, comparison of these populations provides replicate population measures of differential thermal tolerance for each species. Collection from these two locations was chosen because there is evidence of differentiation between the east and west populations for both species. Carson (1996) demonstrated that east and west populations of *D. silvestris* are two different races, with different numbers of rows of bristles on the front legs of males in the two races that are used in courtship displays. There is also evidence that *D. sproati* located on the east and west side of the island are genetically distinct populations (Eldon *et al*., 2013).

Maintenance of wild-caught *Drosophila* and their subsequent progeny

Drosophila silvestris and *D. sproati* are long-lived *Drosophila*, with generation times ~3 months and adults reaching sexual maturity at approximately 1.5–2 weeks post-eclosion for males and 3 weeks post-eclosion for females when raised in laboratory conditions at 16–18°C, standard temperatures for raising Hawaiian picture-wing *Drosophila* (Craddock and Boake, 1992; Boake and Hoikkala, 1995). In the laboratory, the wild-caught flies were used to initiate laboratory populations reared in a common controlled-environment room. The flies were cultured at 16–17°C with 70% humidity and 13 h–11 h light–dark cycle. Forty-three *D. sproati* (20 males and 23 females) and 30 *D. silvestris* (16 males and 14 females) from the SR population and 36 *D. sproati* (17 males and 19 females) and 15 *D. silvestris* (seven males and eight females) from the SKFR population were used as breeders to initiate the laboratory populations. Flies were allowed to breed in 3.78 litre glass jars containing moist sand. Oviposition occurred in vials containing Wheeler–Clayton medium and a folded moist paper towel coated with Olapa tea made from distilled water and the crushed bark of the host plant, Olapa. The larvae were provided with a standard cornmeal medium with dry yeast three times per week. Vials with third instar larvae were placed in glass emerging jars containing moistened coarse sand, which larvae use to burrow and pupate. Emerging adult flies crawl back out after eclosion. Adults were aspirated and sexed between 1 and 7 days after emergence using light CO_2 anaesthesia. Adult female and male flies were separated and placed into 1.89 and 0.94 litre glass jars with moist sand and adult-food vials. Flies used in the experiments were bred within two or three generations after collection from the wild to prevent confounding factors such as age, food type, acclimation to temperatures in the wild and maternal effects and to avoid prolonged laboratory adaptation. The adult flies used in heat- and cold-tolerance experiments were 3–4 weeks post-eclosion, which is a typical age for conducting phenotypic studies of adult Hawaiian picture-wing *Drosophila* (Boake and Hoikkala, 1995; Price *et al*., 2014).

Adult survival after heat treatment

The heat-stress experimental design involved placing 8–10 adult flies in 5 cm × 5 cm × 2 cm transparent plastic chambers, with males and females in separate chambers. After the adult flies had been placed in the chambers, they were moved to a covered, insulated water bath at 32.5°C for 2 h. The number of individuals awake (walking or standing up) and knocked out (KO; those on their backs at the bottom of the box) was recorded. Between 24 and 30 flies in three different

chambers were observed for each combination of species, population and sex.

Sperm mobility after heat-treatment experiment

Sperm mobility was scored in 3- to 4-week-old sexually mature virgin male flies to ensure that the males were fully mature (Craddock and Boake, 1992). Each male was placed in a Petri dish (3.5 cm × 1.5 cm) that was lined with filter paper and then placed in a water bath with a water temperature of 30 (treatment) or 16°C (control) for 1 h. There were up to 10 males observed for each combination of species and population. The high-temperature treatment for the sperm mobility experiment was set below the adult survival heat-treatment temperature because male fertility has been shown to be more sensitive to high-temperature stress (Hoffmann, 2010). The sperm mobility of each male was determined following a protocol slightly modified from Chakir *et al.* (2002). After each male had been dissected, the testes were placed in 20 µl of *Drosophila* Ringer solution (182 mM KCl, 46 mM NaCl, 3 mM $CaCl_2$ and 10 mM Tris–HCl) on a microscope slide. A coverslip was placed over the testes and, after the seminal vesicle had been visually located, slight pressure was applied to the coverslip, causing the seminal vesicle to break open. The testes were viewed within 3 min of dissection at ×100 magnification under a compound microscope, and sperm mobility was quantified using the following scoring system: 0 for no visible sperm moving; 1 for <5% of the sperm observed moving; 2 for >5% but <80% of the sperm observed moving; and 3 for >80% of the sperm observed moving.

Chill-coma recovery

The chill-coma recovery protocol, modified from Sarup *et al.* (2009), involved placing each mature fly, 3–4 weeks old, in a separate Petri dish (3.5 cm × 1.5 cm). The flies in the Petri dishes were then placed on top of a slushed-ice bath, in which the air temperature was 2°C. The Petri dishes containing the flies were removed from the slushed-ice bath after 1.5 h and placed in the controlled-temperature room (16–17°C), where the flies were observed for 60 min. Those flies alive (standing upright) 1–3 min after removal from the slushed-ice bath were recorded as 'survived' and those remaining on their backs were counted as 'knocked out' as they fell into a chill coma. All flies recovered from the chill coma after 60 min. A total of 30–31 flies were measured for chill-coma recovery for each combination of sex, species and population.

Gene expression after heat-treatment experiment

The protocol used for heat treatment in gene expression assays was derived from Sarup *et al.* (2009) and Sørensen *et al.* (2001). Mature adult male flies, 3–4 weeks old, were subjected to a high-temperature treatment at 25°C or control temperature at 16°C for 1 h in a covered, insulated water bath. The lower temperature of 16°C was chosen to reflect typical high temperatures in the wild where the original populations were collected. The high-temperature treatment of 25°C for the gene expression experiment was chosen to reflect a temperature that was slightly above the typical temperatures found in the wild at these locations but could occur in the near future due to climate change in Hawaii. Each fly was aspirated into an individual Petri dish (3.5 cm × 1.5 cm) lined with filter paper. After 1 h of exposure to heat or control temperature, each fly was aspirated into an RNAse-free 1.5 ml microcentrifuge tube and flash frozen in liquid nitrogen followed by storage in a −80°C freezer.

Total RNA extraction

Total RNA was extracted with NucleoSpin® RNA II Kit with DNAse treatment from Macherey Nagel GmbH & Co. KG, Germany. Whole frozen flies were homogenized manually with an RNAse-free microcentrifuge pestle in a 1.5 µl tube containing 350 µl of RA buffer 1 and 3.5 µl of β-Mercaptoethanol. The RNA was eluted with 40 µl of double-distilled water. The concentration of RNA was measured using NanoDrop Spectroscopy (Thermo Scientific, Washington, DE, USA).

Microarray analysis

Probes for the Agilent microarray slides were made using a closely related species, *Drosophila grimshawi* genome (Agilent Technologies, Santa Clara, CA, USA). There was a total of six to eight arrays on eight slides, and the individual samples from different species, populations and treatments were randomized on the arrays to avoid any systematic bias that might occur between locations on the microarray slides. The RNA from a total of 62 individuals was extracted for this experiment, with six to 10 males from each population, species and treatment. After extraction of total RNA, the samples were normalized to a concentration of 30 ng/µl and kept frozen at −80°C until shipment to John A. Burns School of Medicine at the University of Hawaii at Manoa core genetics facility for subsequent reverse transcription to cDNA and microarray analysis. The low-input quickAmp Labeling kit (Agilent Technologies) protocol was closely followed. The labelled samples were then hybridized into the Agilent microarray slides (Agilent Technologies) overnight at 65°C. The next day, the microarray slides were washed at room temperature (37°C) for 1 min each using Agilent wash buffers, after which the microarray slides were scanned using Agilent scanner G2565CA. The intensities of the adherent dye were recorded using Feature extraction software (Agilent Technologies).

Microarray bioinformatics

The initial array was not annotated for use on *D. sproati* and *D. silvestris*. The workflow in Fig. 1 was used to associate Gene Ontology (GO) annotations with the array probes, detect differential expression and identify GO terms that where enriched between the differentially expressed genes. The commercial array contained 14 850 probes, of which 14 792 had previously been mapped to *D. grimshawi* Flybase transcript identifiers. These were converted to Flybase Gene IDs using the

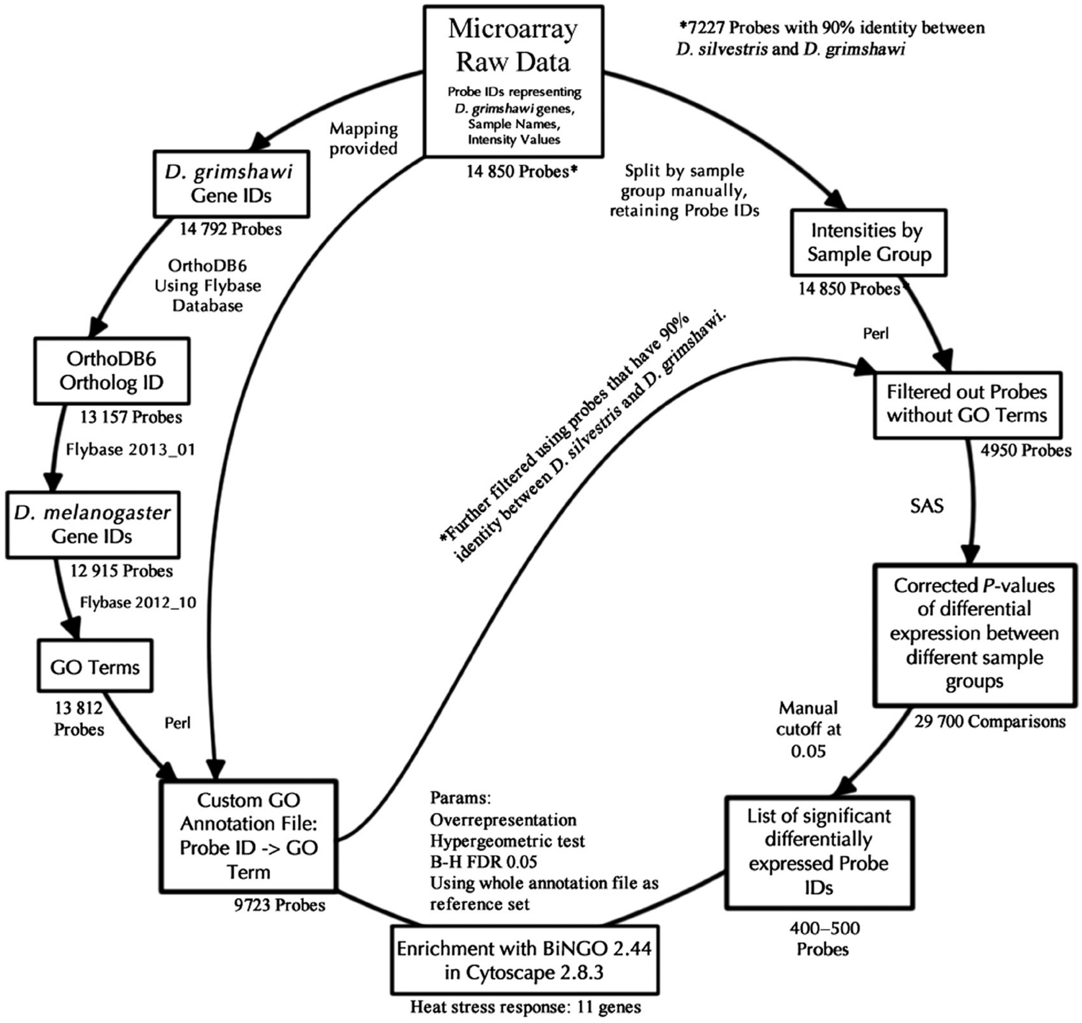

Figure 1: Bioinformatics workflow used to detect enriched Gene Ontology (GO) terms between 16 and 25°C treatments of Hawaiian picture-wing *Drosophila*. *Drosophila grimshawi* GO annotations were associated with the microarray probes using OrthoDB6. These values were then associated with array intensities, and a hierarchical linear model was used to generate the list of significant differentially expressed probes. These results were used in BiNGO to identify enriched GO terms. * The analysis was done with the original 14 792 probes and with the 7227 probes that had 90% identity between *D. silvestris* and *D. grimshawi*.

Flybase ID Conversion tool (http://flybase.org/static_pages/downloads/IDConv.html). Of these, 13 157 could be mapped with OrthoDB E06 (Flybase 2013_01) to *D. melanogaster*, and 12 915 of these *D. melanogaster* gene identifiers could be associated with GO terms. At the time of analysis, 13 812 *D. melanogaster* genes were annotated with at least one GO term. This resulted in a custom GO annotation file with 9723 GO annotated probes. Each probe was filtered to identify those probes on the array that could be GO annotated using the custom GO annotation file. Filtering was done for sequences with a 90% identity between identical sequences in *D. silvestris* and *D. grimshawi* (L. Kang, R. Settlage, W. McMahon, K. Michalak, E. Stacy, D. Price and P. Michalak, unpublished data). Independently, probe intensities were calculated by sample group. Table 1 lists the number of separate arrays for which intensities were determined. This resulted in 4950 probes for which there was an intensity reading for each sample and for which GO annotations could be assigned.

Table 1: Number of separate arrays by species, population and temperature

	16°C	25°C
Drosophila silvestris, SKFR	8	8
D. silvestris, SR	7	9
Drosophila sproati, SKFR	7	10
D. sproati, SR	6	7

Abbreviations: SKFR, South Kona Forest Reserve; SR, Saddle Road.

Statistical analyses

Statistical analyses of adult heat-stress survival and cold-stress survival were computed using binary logistic statistics, with species (*D. silvestris* or *D. sproati*), population location (SKFR or SR) and sex (male or female) as main factors along with the interaction terms between the main factors in the model. Individuals that survived were counted as 1 and those that did not survive as 0. Statistical analyses of sperm mobility were computed using ordinal logistic regression, with species and population as main factors and the interaction between species and population location in the model. Both binary and ordinal logistic regression analyses were computed using the statistical program MiniTab® version 16 (Minitab Inc., State College, PA, USA). Statistical significance for the microarray data was computed as follows. For each species, the 4950 GO annotated probe intensities were compared between temperature levels using hierarchical linear models (proc mixed in SAS, The SAS Institute Cary, NC, USA; SAS scripts are available in the Supplemental data). For each species, for each probe, there was an analysis to test for a fixed difference in expression level between the two temperature levels, considering each site as a random factor and each array as an independent measure. The results for each species were corrected according to Benjamini and Hochberg (1995). This resulted in 246 differentially expressed GO annotated probes for *D. sproati* and 106 for *D. silvestris*. These were then analysed for GO enrichment using BiNGO 2.44 within Cytoscape 2.8.3. Over-representation of GO terms was determined using a Benjamini and Hochberg 0.05 false discovery rate (FDR) corrected hypergeometric test.

Results

Adult survival after heat-treatment experiment

Overall, the binary logistic regression analysis for the full model with species, population and treatment and all two-way and three-way interactions for the proportion of adult survivors in the heat-tolerance experiment was highly significant (Fig. 2A). The results show that *D. sproati* recovered from heat stress at 32.5°C significantly better than *D. silvestris* (*D. silvestris*, $\bar{x} = 0.25$, 1 SEM = 0.0367, $n = 140$; and *D. sproati*, $\bar{x} = 0.76$, 1 SEM = 0.0370, $n = 132$; $P = 0.021$). A significantly higher mean proportion of *D. sproati* females and males than *D. silvestris* females and males survived after heat stress ($P = 0.021$, $n = 272$). There was a significant species-by-population interaction ($P = 0.036$, $n = 272$), owing mostly to the difference in the mean proportion alive from the two *D. sproati* populations, with the SR population having slightly lower survival than the SKFR population. The proportion of *D. silvestris* from SR that survived was not significantly different from that of *D. silvestris* from SKFR ($P = 0.427$). The proportion of adults that survived differed significantly between test dates ($P = 0.031$, $n = 272$). The three-way interaction of species by population by sex was not significant ($P = 0.826$, $n = 272$).

Sperm mobility after heat-treatment experiment

The results of the ordinal logistic regression analysis on sperm mobility scores for the full model with species, population and treatment and all two-way and three-way interactions was highly significant (Fig. 2B). The main results show significantly less mobile sperm after heat treatment at 30°C for both species (16°C, $\bar{x} = 2.03$, 1 SEM = 0.182, $n = 34$; and 30°C, $\bar{x} = 1.30$, 1 SEM = 0.181, $n = 43$; $P = 0.010$). For heat-treated individuals, *D. sproati* had slightly more mobile sperm than *D. silvestris* ($P = 0.064$, $n = 43$). In fact, the sperm mobility score for *D. sproati* (16°C, $\bar{x} = 2.231$, 1 SEM = 0.257, $n = 13$; and 25°C, $\bar{x} = 1.905$, 1 SEM = 0.257, $n = 21$) after heat stress was closer to control scores than the scores for *D. silvestris* (16°C, $\bar{x} = 1.905$, 1 SEM = 0.248, $n = 21$; and 25°C, $\bar{x} = 0.727$, 1 SEM = 0.188, $n = 22$). There was no significant difference between SR and SKFR populations ($P = 0.835$). None of the two-way interactions or three-way interaction between species, population and treatment was significant for sperm mobility ($P = 0.229$–0.903).

Chill coma recovery experiment

The results of the binary logistic regression analysis and mean proportion of adult flies that survived the cold treatment for the full model with species, population and sex and all two-way and three-way interactions was highly significant (Fig. 2C). The main results show a significant difference in cold-stress survival between the two species. More *D. silvestris* were alive and moving after the cold treatment compared with *D. sproati*. There was no significant difference in cold-stress survival between sexes ($P = 0.574$). None of the two-way interactions and three-way interaction between species, population and sex was significant for cold-stress survival ($P = 0.111$–0.298).

Microarray analysis

The overall change in gene expression for both *D. sproati* and *D. silvestris* for individuals exposed to a high temperature (25°C) in comparison to individuals at the control temperature (16°C) indicates that both species had significantly altered gene expression patterns (Fig. 3A and B). Overall, there was a strong positive correlation between the difference in gene expression in *D. sproati* and *D. silvestris* at these two temperatures for all genes (Fig. 3C; $F = 4038.1$; d.f. = 1, 14 850; $P < 0.0001$). The GO analysis showed a total of 13 GO terms to be enriched between temperatures in *D. silvestris* (Fig. 4A–C) compared with only three in *D. sproati* (Fig. 4D). In Tables 2 and 3, the GO terms are listed with statistically significant tests for over-representation between 16 and 25°C samples. All ANOVA calculations were done using SAS proc mixed (SAS Institute, Cary, NC, USA) with restricted maximum likelihood estimations, and

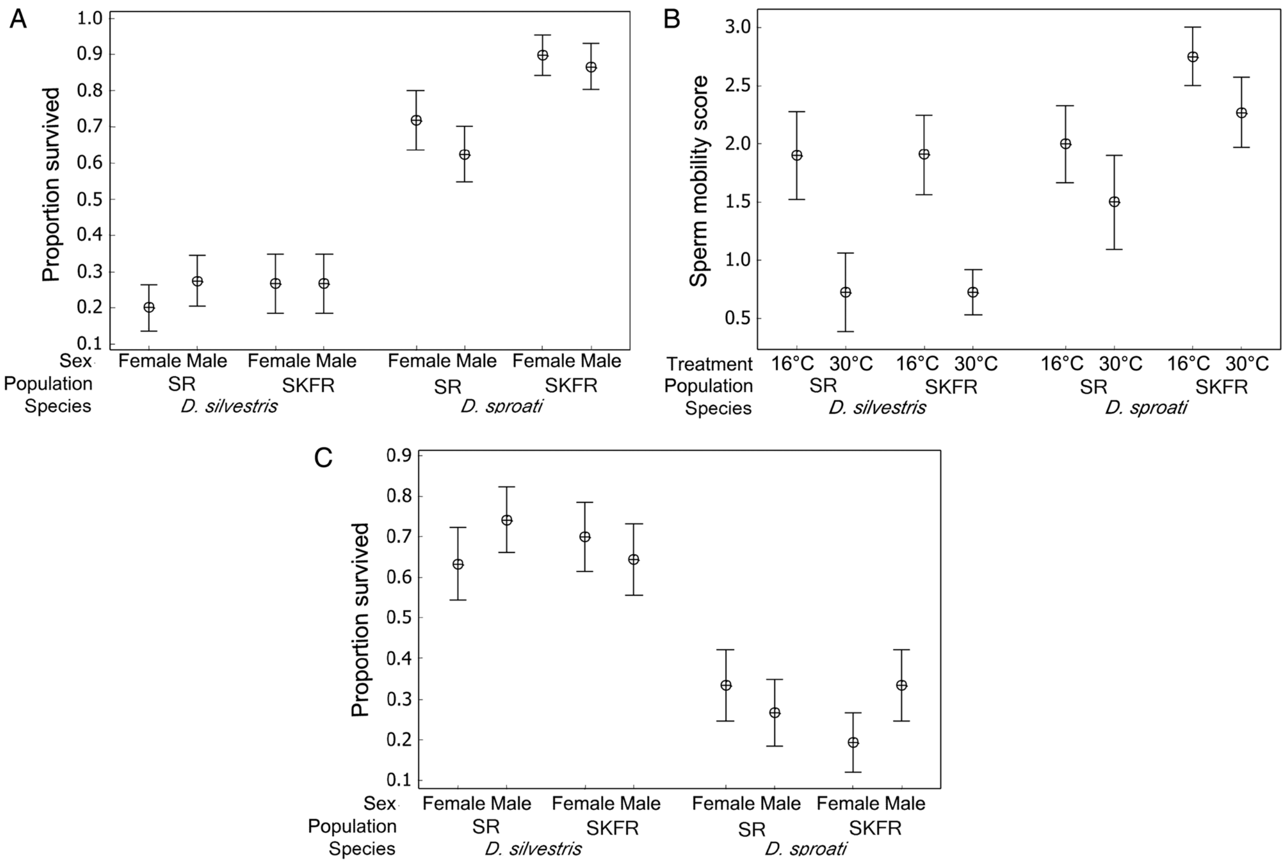

Figure 2: The temperature tolerance of *D. silvestris* and *D. sproati*. (**A**) Proportion of Hawaiian picture-wing *Drosophila* that survived after heat stress for males and females from the Saddle Road (SR) and South Kona Forest Reserve (SKFR) for each species. Flies that were standing and walking after exposure were counted as survivors. More *D. sproati* survived the heat stress compared with *D. silvestris* (overall model, $G = 91.116$, d.f. = 9, *P*-value < 0.001, $n = 272$). (**B**) Sperm mobility scores of sexually mature males from both populations in control (16°C) and heat (30°C) treatments. Sperm from *D. sproati* were more mobile than *D. silvestris* sperm after heat treatment (overall model, $G = 29.171$, d.f. = 8, *P*-value < 0.001, $n = 77$). (**C**) Proportion that survived after cold stress at 2°C for 1.5 h (overall model, $G = 43.088$, d.f. = 7, *P*-value < 0.001, $n = 243$). Flies that were standing and walking after exposure were counted as survivors. More *D. silvestris* survived the cold shock compared with *D. sproati* (*D. silvestris*, $\bar{x} = 0.68$, 1 SEM = 0.0424, $n = 122$; and *D. sproati*, $\bar{x} = 0.28$, 1 SEM = 0.0410, $n = 121$; $P = 0.022$). The mean values ± 1 SEM are reported.

type 3 sums of squares. A hierarchical linear model was used to test for differences in mean probe intensity between the two temperatures for each species, while allowing each sample site to have its own overall mean. Each *P*-value of the resulting 4950 *D. silvestris* and *D. sproati F* tests was corrected for multiple testing with an FDR of 0.05 (Benjamini and Hochberg, 1995). In total, 246 *D. silvestris* and 106 *D. sproati* probes were significantly different between temperature levels with an FDR of 0.05. The significant GO terms for *D. sproati* were primarily associated with protein folding (e.g. heat-shock proteins) and cellular metabolism. Surprisingly, *D. silvestris* had a substantially greater number of significant GO terms with genes associated with uridine kinase activity, regulation of translation processes, regulation of ribosome assembly and protein folding. These gene expression results are consistent with physiological stress experiments indicating that *D. silvestris* exhibits a greater heat stress response than *D. sproati*. Supplemental Tables S1 and S2 contain the ANOVA results for all GO annotated probes analysed.

Discussion

The results reported in this paper indicate that temperature tolerances differ in the laboratory for several traits between two Hawaiian picture-wing *Drosophila* species. The more common species, *D. sproati*, exhibited greater tolerances to high temperature, while the less common species, *D. silvestris*, exhibited reduced tolerance to high temperature and greater tolerances to cold temperature. The temperatures used in this study were cooler than temperatures typically used in experiments with other *Drosophila* species (Sørensen *et al*., 2001; Sarup *et al*., 2009) and reflect the relatively cool temperatures where these species are found in the upper elevation (1400–1500 m) Hawaiian wet rainforests (see Materials and methods; Giambelluca *et al*., 2014). The differences in

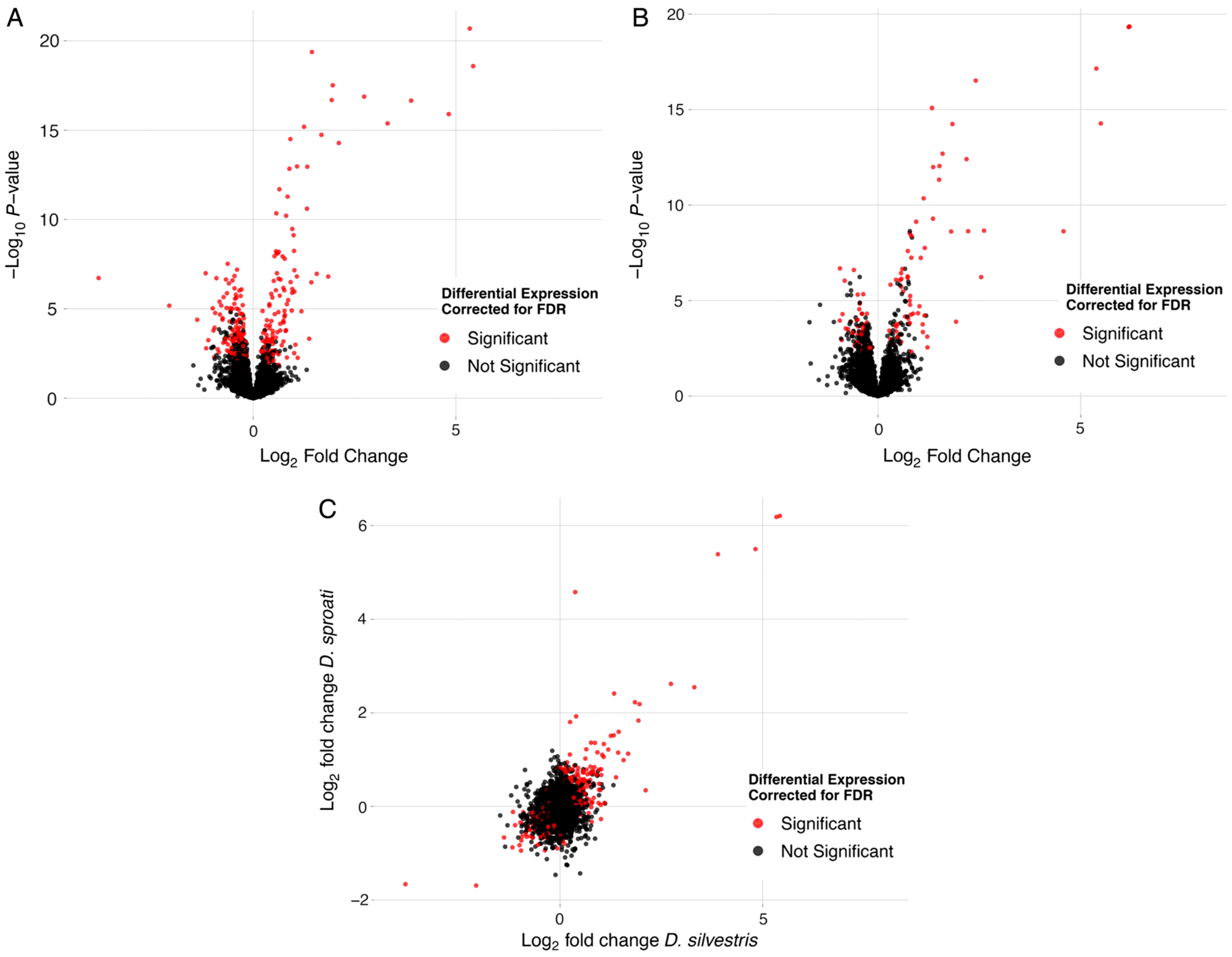

Figure 3: Volcano plots from microarray gene expression analysis for *D. silvestris* males (**A**) and *D. sproati* males (**B**). The *x*-axes on the volcano plots are the $\log_2$ fold-change of the differences in gene expression for flies exposed to 25°C for 1 h compared with control flies exposed to normal temperature for these species at 16°C for 1 h. The *y*-axes show the negative of the $\log_{10}$ corrected *P*-values (Benjamini and Hochberg, 1995) of the comparison of the two treatments for the same microarray probe. (**C**) Scatterplot of the correlation between the $\log_2$ fold-change in gene expression between heat-treated (25°C) and control (16°C) *D. silvestris* and *D. sproati* males.

temperature tolerance found in this study between these two species suggest that *D. sproati* may be a relatively warmer adapted species and *D. silvestris* may be a colder adapted species. The results are consistent with the wider elevation range where *D. sproati* is currently found on Hawaii Island in comparison to *D. silvestris* (D. K. Price, personal observation).

In the heat-stress experiment, both female and male *D. sproati* were found to be more heat tolerant at 32.5°C than the sympatric congenera, *D. silvestris*. Likewise, in the sperm mobility test at 30°C for 1 h, *D. sproati* males from both populations had significantly higher sperm mobility scores than *D. silvestris* males (Fig. 2A and B). Fertility traits may be especially sensitive to temperature changes because the upper temperature limits for these traits typically occur below the temperature range that causes mortality (Hoffmann, 2010). The differences in sperm mobility may indicate that overall fertility of male *D. silvestris* could be affected by higher temperatures, which may limit their distribution. In the cold-stress survival experiment, *D. silvestris* was significantly more tolerant to cold temperatures than *D. sproati* (Fig. 2C). This is consistent with the higher elevation and thus colder environments, where *D. silvestris* is currently found in nature. These differences in temperature tolerance were determined by performing replicated experiments involving two natural populations of both *D. sproati* and *D. silvestris*. Interestingly, there was little difference in the traits measured between populations, which suggests that there has been little evolutionary change between populations in these traits despite the other differences between these two populations for both species (Carson, 1996; Eldon *et al.*, 2013).

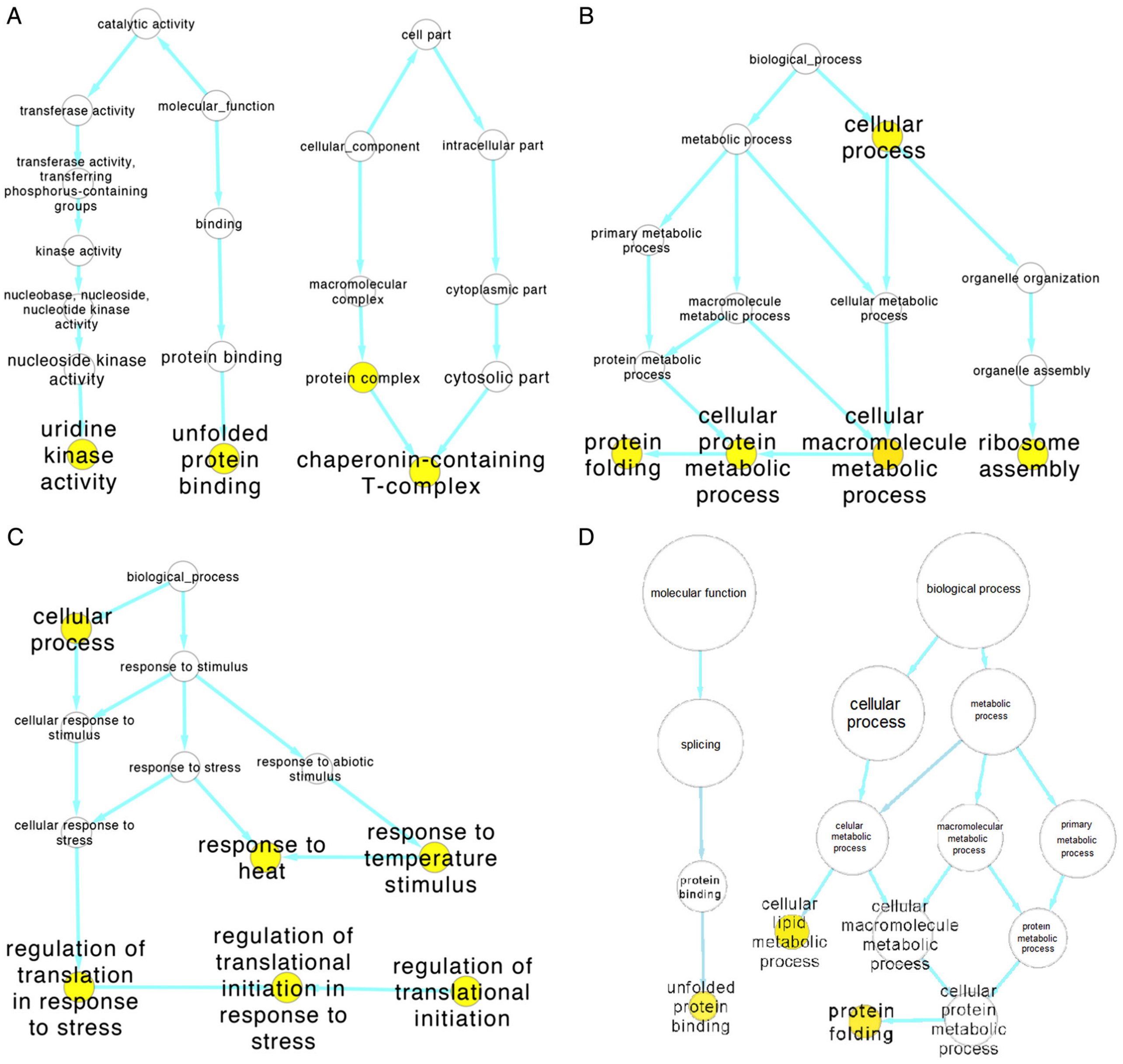

Figure 4: The relationship between GO terms enriched in the annotated microarray probes between 16 and 25°C treatments. Yellow filled circles are terms that are enriched in the results with an FDR of <0.05, while open circles represent non-enriched terms that connect the enriched terms within a cluster. (**A**–**C**) Results for *D. silvestris*. (**A**) Two clusters under the molecular function root term. (**B** and **C**) More complex biological process clusters. (**D**) Relationship of the three significantly enriched GO terms in *D. sproati*.

Table 2: Over-represented Gene Ontogeny terms in *D. sproati*

Description	Number of genes in test set	GO-ID	*P*-Value	Corrected *P*-value	*x*	*n*	*X*	*N*
Protein folding	21	6457	1.80×10^{-6}	5.26×10^{-3}	21	122	510	9499
Unfolded protein binding	15	51 082	1.88×10^{-5}	2.75×10^{-2}	15	80	510	9499
Cellular lipid metabolic process	24	44 255	4.63×10^{-5}	4.51×10^{-2}	24	184	510	9499

Abbreviation: GO-ID, gene ontology identification.

Table 3: Over-represented Gene Ontogeny terms in *D. silvestris*

Description	Number of genes in test set	GO-ID	*P*-Value	Corrected *P*-value	*x*	*n*	*X*	*N*
Cellular macromolecule metabolic process	105	44 260	1.15×10^{-6}	2.72×10^{-3}	105	1759	368	9499
Cellular process	237	9987	1.40×10^{-5}	1.41×10^{-2}	237	5099	368	9499
Protein folding	16	6457	1.80×10^{-5}	1.41×10^{-2}	16	122	368	9499
Unfolded protein binding	12	51 082	5.32×10^{-5}	3.13×10^{-2}	12	80	368	9499
Response to temperature stimulus	13	9266	8.73×10^{-5}	3.98×10^{-2}	13	97	368	9499
Cellular protein metabolic process	68	44 267	1.12×10^{-4}	3.98×10^{-2}	68	1128	368	9499
Response to heat	11	9408	1.18×10^{-4}	3.98×10^{-2}	11	74	368	9499
Chaperonin-containing T-complex	4	5832	1.37×10^{-4}	4.03×10^{-2}	4	8	368	9499
Regulation of translational initiation in response to stress	3	43 558	2.24×10^{-4}	4.33×10^{-2}	3	4	368	9499
Regulation of translation in response to stress	3	43 555	2.24×10^{-4}	4.33×10^{-2}	3	4	368	9499
Uridine kinase activity	3	4849	2.24×10^{-4}	4.33×10^{-2}	3	4	368	9499
Ribosome assembly	3	42 255	2.24×10^{-4}	4.33×10^{-2}	3	4	368	9499
Regulation of translational initiation	4	6446	2.39×10^{-4}	4.33×10^{-2}	4	9	368	9499

Abbreviation: GO-ID, gene ontology identification.

Overall, these results indicate that *D. sproati* may be more tolerant to higher temperatures, which could suggest that they may be able to breed in the wild even during relatively hot days and at lower elevations than *D. silvestris*. Laboratory studies have shown success in producing more heat-tolerant *Drosophila* populations compared with control lines within five generations of lines selected for thermal resistance (Parsons, 1989). Hoffmann (2010) argued that heritability in heat-stress-resistant traits is low in the laboratory setting (10–20%) and less likely to be experienced in the field. He also suggested that in some populations, genetic variation may not be enough to counter physiological limits because of strong trait and gene interactions. If species have limited ability to evolve heat tolerance, this could suggest that *D. silvestris* will not be able to adapt easily to increasing temperatures. Future studies on thermal stress resistance of female fertility, such as number of eggs laid after heat stress, should also be carried out. This will shed light on the potential impacts in natural populations on female reproduction after exposure to future warmer temperatures.

The high temperatures of 30.0 or 32.5°C are not likely to occur in the near future where these species are found because the current average air temperature in the area is 12.8–14.4°C, with a high of 25.8°C and low of 0.0°C (Giambelluca *et al.*, 2014). However, knowing that *D. sproati* can survive such heat stress for a short length of time (i.e. during hottest days of the year) indicates that they may be better able to withstand higher than normal temperatures brought about by climate change. These differences in heat tolerance could be compounded with other ecological threats, such as predation by the introduced yellow jacket wasp, which might disproportionally affect *D. silvestris* once it is slowed down by heat stress (Foote and Carson, 1995). The *D. silvestris* and *D. sproati* populations are less likely be impacted negatively by host-plant distributions than some other Hawaiian picture-wing *Drosophila* because they both use the same host plant, Olapa (*C. trigynum*), which is a common understory tree in the wet rainforests on Hawaii Island (Magnacca *et al.*, 2008). *Drosophila silvestris* also uses plants in the genus *Clermontia*, of which there are many currently rare species. It is not clear how the various ecological factors may combine to limit the distribution and abundances of the different Hawaiian *Drosophila* species.

Heat tolerance of Hawaiian *Drosophila* during other life stages could also be important in their adaptation to climate change. Natural temperatures at the egg, larval and pupal stages are likely to fluctuate less than during the adult stage because eggs and larvae for these Hawaiian picture wings live in decaying bark and pupae are in the soil and leaf litter, which should be more buffered from changes in temperature. However, given that these life stages are less mobile they may be more prone to heat stress because, unlike adults, they have limited abilities to thermoregulate behaviourally during hot weather. Depending on the intensity and length of hotter days in the future, studies are needed to address the importance of fluctuating temperatures for different life stages.

Gene expression

What mechanisms might allow *D. sproati* to tolerate higher temperatures than *D. silvestris*? An answer may be that heat tolerance is gene related or partly due to the expression level of heat-shock proteins, other metabolic or other gene products. Higher levels of *hsp70* expression in adult *D. buzatii* were

related to a higher intensity of stress and less tolerance to heat (Sørensen *et al.*, 2001). Experimentally increasing copies of *hsp70* slowed the growth and development of *D. melanogaster* (Krebs and Feder, 1997). Therefore, the amount of *hsp70* expression in the *Drosophila* could directly result in the ability to survive or tolerate heat stress.

In this study, *D. sproati* and *D. silvestris* males were subjected to a mild temperature increase to 25°C or left at the control temperature of 16°C for 1 h, and gene expression profiles were analysed using Agilent microarray slides. Those microarray probes that could be annotated with GO functionality were used for detecting GO enrichment. Figure 3 shows the relationship between GO terms enriched in the set of genes with statistically significant differences for the temperature main effect, with an FDR = 0.05. In other words, several of these GO terms appear more frequently in the set of annotated probes showing a temperature main effect then would be predicted by chance alone. For *D. silvestris*, there is clearly a response in protein metabolism (Fig. 4A and B), and much of this response appears to be a stress response (Fig. 4C). The response in *D. sproati* is much less pronounced (Fig. 4D). The microarray results revealed a suite of heat-shock protein genes very highly increased in expression after mild heat stress. The most upregulated gene was *hsp70*, which had a 5-fold increase in expression at 25 compared with 16°C and indicates that both species may have experienced heat stress at 25°C. *Drosophila silvestris* showed a slightly greater change between temperature treatments in *hsp70* expression compared with *D. sproati*. This is consistent with other research on *D. buzatii* thermal tolerance, where *hsp70* expression was higher for populations least tolerant to stress. In the *D. buzatii* experiment, flies that survived better after heat shock at 41°C also expressed *hsp70* at lower levels after exposure to 37 and 39°C (Sørensen *et al.*, 2001). *hsp70* expression is an important defence mechanism in cells. Heat stress, metal contaminants, cold stress and oxidative stress result in the misfolding of proteins, and *hsp70* binds to these misfolded proteins to prevent further unfolding and aggregation that would otherwise cause apoptosis. Small amounts of *hsp70* are beneficial to organisms because they prevent unwanted cell death, but overexpression of this protein could cost the organism growth and development activity (Krebs and Loeschcke, 1994; Kregel, 2002). Therefore, priority to produce *hsp70* and other heat-shock proteins instead of normal cell activity, in conjunction with apoptosis, may be one cause of the lower survival in *D. silvestris* after heat shock.

A surprising result from this study is the differences in the change in gene expression between these two species at this mildly elevated temperature (25°C) compared with the control temperature (16°C). Growth, development and immune response genes were upregulated in *D. sproati* but downregulated in *D. silvestris*. In contrast, metabolic activity and protein transport were upregulated in *D. silvestris*. This suggests that *D. sproati* is only mildly affected by the 25°C heat stress, because *D. sproati* individuals were able to focus their energy on growth and development while *D. silvestris* individuals were making more *hsp70* and may only have been able to process metabolic activity to produce enough energy for the heat-shock response. A study on *D. melanogaster* females showed physiological conditioning, which occurs when flies are exposed to mild heat stress several times during the insect's lifespan, resulting in increased ability to tolerate hotter temperatures, but at the same time, decreased ability to produce eggs (Krebs and Loeschcke, 1994). It would be valuable to measure reproductive ability and population viability across multiple generations in combination with gene expression experiments at the mildly increased temperature of 25°C that is likely to occur in the near future in many of these mid-elevation habitats in Hawaii. This could determine the relationship between the changes in different sets of genes with important survival and reproductive traits at temperatures relevant for natural populations (Sørensen, 2010).

Two genes that were highly upregulated in *D. sproati* after heat stress but not in *D. silvestris* were hsp26 and hsp22. Experimental evidence demonstrates that the function of these small heat-shock proteins is related to the response to heat and the determination of adult lifespan (Tweedie *et al.*, 2009). In general, small heat-shock proteins are involved in preserving actin and cytoskeletal structures as well as preventing capsase-dependent apoptosis (Colinet *et al.*, 2010). Unlike other small heat-shock proteins, hsp26 is activated only during elevated temperatures (Haslbeck *et al.*, 1999). The expression of hsp26 and hsp22 in high amounts in *D. sproati* may be the reason for their greater resistance to heat stress. In *D. melanogaster*, elevated *hsp70* expression has been shown to reduce developmental abnormalities associated with temperature stress (Roberts and Feder, 1999), and it may be possible that these small heat-shock proteins operate in conjunction with *hsp70* to promote normal expression and regulation of other genes and mediate normal cell activity and function, permitting *D. sproati* to tolerate higher temperatures.

It is possible that some of the apparent differences in gene expression between *D. silvestris* and *D. sproati* found in this study may be due differences in cross-species hybridization because the microarray probes were designed using *D. grimshawi* genome (Bar-Or *et al.*, 2006). *Drosophila grimashawi*, the only Hawaiian picture-wing *Drosophila* species that has been sequenced to date (Drosophila 12 Genomes Consortium *et al.*, 2007), is more closely related to *D. sproati* than to *D. silvestris* (Magnacca and Price, 2012; K. M. Magnacca and D. K. Price, unpublished data). This might suggest that additional genes that were not detected in this study may be important in temperature tolerances in *D. silvestris* or *D. sproati*. Another potential concern in cross-species hybridization studies is changes in binding ability of cDNA from one or both of the species to the microarray probes (Bar-Or *et al.*, 2006). It might be expected that *D. silvestris* would show fewer changes in gene expression due to the greater evolutionary time for changes to accumulate in regions of the genes that impact binding to the probes. However, *D. silvestris* exhibited more gene expression changes, 246 genes and 13 GO terms, compared with *D. sproati*, 106 genes and three GO terms. In other studies of cross-species hybridization of microarray probes,

there has not been a correspondence between the hybridization of probes and genetic relatedness of the species (Bar-Or *et al.*, 2006). Future studies that examine specific candidate genes or genome-wide RNA-sequencing experiments could address this concern and potentially identify additional genes or GO terms to be important in temperature tolerance in these species.

Drosophila are useful monitors of environmental change. They are widely distributed and relatively easy to collect in nature, many can be raised in the laboratory, and they can be good indicator species of forest degradation (Parsons, 1989; Hoffmann, 2010). On a continental scale, gene arrangements associated with warm climate are increasing in frequency in populations of *D. pseudoobscura* in both the Americas and Europe, indicating that there is genetic variation and evolution of temperature tolerance (Rezende *et al.*, 2010). Comparison of *Drosophila* species from different regions of the world indicates that both heat and cold tolerance may be due to adaptation to climate differences at the location of species origin (Hoffmann *et al.*, 2003). In Hawaii, Foote and Carson (1995) first noted the decline in species diversity of Hawaiian picture-wing *Drosophila* in the Olaa Rainforest on Hawaii Island. They observed several species that declined in abundance and are currently extinct locally, including *D. silvestris*, and several other species that increased in relative abundance, including *D. sproati*. The decline in abundance and occurrence of some Hawaiian *Drosophila* species may be due to several factors, namely degradation of forests by ungulates, predation by the introduced yellow jacket wasp (*Vespula pennsylvanica*), decline of host plants and/or competition with cosmopolitan species. It is also possible that climate change is contributing to the change in species diversity of the Hawaiian *Drosophila*. Recent reports indicate that temperature has increased over the last 30 years in Hawaii, especially at higher elevations (Giambelluca *et al.*, 2008). It is not clear whether the temperature changes that have occurred in Hawaii could lead to changes in the distribution and abundances of Hawaiian *Drosophila* or ectothermic animals in general. The results from this study, showing greater heat tolerance of *D. sproati*, are consistent with the increased abundance in some habitats of *D. sproati* and reduced abundance of the less heat-tolerant *D. silvestris*.

Future studies should focus on determining the temperature tolerances of the many rare and declining species in comparison with the more common Hawaiian *Drosophila* in order to manage their conservation better, as well as using the observations of different species as an indicator of change in temperature in the environment. The Hawaiian picture-wing *Drosophila* living in high-elevation wet forest habitat may experience changes in distribution because many of them live in similar wet forest or mesic forest habitat (1200 m and above). The Hawaiian picture-wing *Drosophila* are connected to the community-level food webs involving species-specific host plants and other potential species interactions in the Hawaiian forest. The decline in diversity of Hawaiian picture-wing *Drosophila* should continue to be investigated because they are good indicators of the health of the Hawaiian terrestrial environment.

The story does not need to end with the demise of cold-adapted Hawaiian picture-wing *Drosophila*. Out of the approximately 120 picture-wing species (O'Grady *et al.*, 2011; Magnacca and Price, 2012), there is hope for species to survive global warming. The more warm-tolerant picture-wings, such as *D. sproati*, may survive in the mid-elevation habitats, while other species will probably need to move to higher elevations. To promote the longer-term survival of Hawaiian *Drosophila* species, habitat restoration efforts should include establishing host plants of these native insects at higher elevations.

Supplementary material

Supplementary material is available at *Conservation Physiology* online.

Acknowledgements

We thank Anne Veillet at the University of Hawaii at Hilo Core Genetics Facility and Steffen Oeser at the University of Hawaii at Manoa John A. Burns School of Medicine Genomics Core Facility for assisting with the RNA extractions and microarray analyses.

Funding

This research is based upon work supported by the Sigma Xi Grants-in-Aid of Research (GIAR) program and the National Science Foundation under grant numbers EHR-0833211 and EPS-0903833 to the University of Hawaii at Hilo and number ABI-1062432 to Indiana University. Any opinions, findings and conclusions or recommendations expressed in this material are those of the authors and do not necessarily reflect the views of the National Science Foundation, the University of Hawaii at Hilo, National Center for Genome Analysis Support or Indiana University.

References

Asner GP (2013) Mesoscale exploration and conservation of tropical canopies in changing climate. In Lowman M, Devy S, Ganesh T, eds, *Treetops at Risk: Challenges of Global Canopy Ecology and Conservation*. Springer Science & Business Media, New York, pp 177–193.

Atkinson CT, LaPointe D (2009) Introduced avian diseases, climate change, and the future of Hawaiian honeycreepers. *J Avian Med Surg* 23: 53–63.

Bale JS, Masters GJ, Hodlinson ID, Awmack C, Bezmer TM, Brown VK, Butterfield J, Buse A, Coulson JC, Farrar J et al. (2002) Herbivory in global climate change research: direct effects of rising temperature on insect herbivores. *Glob Change Biol* 8: 1–16.

Bar-Or C, Czosnek H, Koltai H (2006) Cross-species microarray hybridizations: a developing tool for studying species diversity. *Trends Genet* 23: 200–207.

Benjamini Y, Hochberg Y (1995) Controlling the false discovery rate: a practical and powerful approach to multiple testing. *J R Statist Soc B* 57: 89–300.

Boake CRB, Hoikkala A (1995) Courtship behaviour and mating success of wild-caught *Drosophila silvestris* males. *Anim Behav* 49: 1303–1313.

Carson HL (1982) Evolution of *Drosophila* on the newer Hawaiian volcanoes. *Heredity* 48: 3–25.

Carson HL (1996) Sexual selection: a driver of genetic change in Hawaiian *Drosophila*. *J Hered* 88: 343–352.

Chakir M, Chafik A, Moreteau B, Gibert P, David JR (2002) Male sterility thermal thresholds in *Drosophila*: *D. simulans* appears more cold adapted than its sibling *D. melanogaster*. *Genetica* 114: 195–205.

Drosophila 12 Genomes Consortium, Clark AG, Eisen MB, Smith DR, Bergman CM, Oliver B, Markow TA, Kaufman TC, Kellis M, Gelbart W et al. (2007) Evolution of genes and genomes on the *Drosophila* phylogeny. *Nature* 450: 203–218.

Colinet H, Lee SF, Hoffmann AA (2010) Temporal expression of heat shock genes during cold stress and recovery from chill coma in adult *Drosophila melanogaster*. *FEBS J* 277: 174–185.

Craddock E, Boake CRB (1992) Onset of vitellogenesis in female *Drosophila silvestris* is accelerated in the presence of sexually mature males. *J Insect Physiol* 38: 643–650.

Craddock EM, Carson HL (1989) Chromosomal inversion patterning and population differentiation in a young insular species, *Drosophila silvestris*. *Proc Natl Acad Sci USA* 86: 4798–4802.

Deutsch CA, Tewksbury JJ, Huey RB, Sheldon KS, Ghalambor CK, Haak DC, Martin PR (2008) Impacts of climate warming on terrestrial ectotherms across latitude. *Proc Natl Acad Sci USA* 105: 6668–6672.

Eldon J (2010) Population divergence in an endemic Hawaiian *Drosophila*: phylogeographic patterns and local adaptation. Unpublished master's thesis, University of Hawaii at Hilo, Hilo, HI, USA.

Eldon J, Price JP, Magnacca K, Price DK (2013) Patterns and processes in complex landscapes: testing alternative biogeography and community ecology in Hawaii. *Mol Ecol* 22: 3613–3628.

Foote D, Carson H (1995) Drosophila as monitors of change in Hawaiian ecosystems. In LaRoe ET, Farris GS, Puckett CE, Doran PD, Mac MJ, eds, *Our Living Resources: a Report to the Nation on the Distribution, Abundance and Health of U.S. Plants, Animals and Ecosystems*. US Department of the Interior, National Biological Service, Washington, DC, USA, pp 368–372.

Giambelluca TW, Nullet MA, Schroeder TA (1986) *Rainfall Atlas of Hawaii*. Department of Land and Natural Resources, Division of Water and Land Department, Hololulu, HI, USA.

Giambelluca TW, Diaz HF, Luke MSA (2008) Secular temperature changes in Hawai'i. *Geophys Res Lett* 35: L12702.

Giambelluca TW, Shuai X, Barnes ML, Alliss RJ, Longman RJ, Miura T, Chen Q, Frazier AG, Mudd RG, Cuo L et al. (2014) Evapotranspiration of Hawai'i. Final report submitted to the U.S. Army Corps of Engineers—Honolulu District, and the Commission on Water Resource Management, State of Hawai'i. http://climate.geography.hawaii.edu/interactivemap.html.

Haslbeck M, Walke S, Stromer T, Ehrndperger M, White HE, Chen S, Saibil HR, Buchner J (1999) Hsp26: a temperature regulated chaperone. *EMBO J* 18: 6744–6751.

Hobbelen PH, Samuel MD, Foote D, Tango L, LaPointe DA (2013) Modeling the impacts of global warming on predation and biotic resistance: mosquitos, damselfies and avian malaria in Hawaii. *Theor Ecol* 6: 31–44.

Hoffmann AA (2010) Physiological climatic limits in *Drosophila*: patterns and implications. *J Exp Biol* 213: 870–880.

Hoffmann AA, Sørensen JG, Loeschcke V (2003) Adaptation of *Drosophila* to temperature extremes: bringing together quantitative and molecular approaches. *J Therm Biol* 28: 175–216.

IPCC (2007) Summary for policymakers. In Solomon S, Qin D, Manning M, Chen Z, Marquis M, Averyt KB, Tignor M, Miller HL, eds, *Climate Change 2007: The Physical Science Basis. Contribution of Working Group I to the Fourth Assessment Report of the Intergovernmental Panel on Climate Change*. Cambridge University Press, Cambridge, UK and New York, NY, USA.

Jokiel PL, Brown EK (2004) Global warming, regional trends and inshore environmental conditions influence coral bleaching in Hawaii. *Glob Change Biol* 10: 1627–1641.

Krebs RA, Feder ME (1997) Deleterious consequences of Hsp70 overexpression in *Drosophila melanogaster* larvae. *Cell Stress Chaperones* 2: 60–71.

Krebs RA, Loeschcke V (1994) Costs and benefits of activation of the heat-shock response in *Drosophila melanogaster*. *Funct Ecol* 8: 730–737.

Kregel KC (2002) Heat shock proteins: moditying factors in physiological stress responses and aquired thermotolerance. *J Appl Physiol* 92: 2177–2186.

Krushelnycky PD, Loope LL, Giambelluca TW, Starr F, Drake D, Taylor A, Robichaux R (2013) Climate-associated population declines reverse recovery and threaten future of an iconic high-elevation plant. *Glob Change Biol* 19: 911–922.

Lauer A, Zhang C, Elison-Timm O, Wang Y, Hamilton K (2013) Downscaling of climate change in the Hawaiian region using CMIP5 results: on the choice of the forcing fields. *J Climate* 26: 10006–10030.

Loope LL, Giambelluca TW (1998) Vulnerability of island tropical montane cloud forests to climate change, with special reference to East Maui, Hawaii. *Clim Change* 39: 503–517.

Magnacca KN, Price DK (2012) New species of Hawaiian picture-wing *Drosophila* (Diptera: Drosophilidae), with a key to species. *Zootaxa* 3188: 1–30.

Magnacca KN, Foote D, O'Grady PM (2008) A review of the endemic Hawaiian *Drosophilidae* and their host plants. *Zootaxa* 1728: 1–58.

O'Grady PM, Lapoint RT, Bonacum J, Lasola J, Owen E, Wu Y, DeSalle R (2011) Phylogenetic and ecological relationships of the Hawaiian *Drosophila* inferred by mitochondrial DNA analysis. *Mol Phylogenet Evol* 58: 244–256.

Parmesan C (2006) Ecological and evolutionary responses to recent climate change. *Annu Rev Ecol Evol Syst* 37: 637–669.

Parsons PA (1989) Conservation and global warming: a problem in biological adaptation to stress. *Ambio* 18: 322–325.

Price DK, Muir C (2008) Conservation implications of hybridization in Hawaiian picture-winged *Drosophila*. *Mol Phylogenet Evol* 47: 1217–1226.

Price DK, Souder S, Russo-Tait T (2014) Sexual selection, epistasis and species boundaries in sympatric Hawaiian picture-winged *Drosophila*. *J Insect Behav* 27: 27–40.

Rezende EL, Balanyà J, Rodríguez-Trelles F, Rego C, Fragata I, Matos M, Serra L, Santos M (2010) Climate change and chromosomal inversions in *Drosophila subobscura*. *J Climate Res* 44: 103–114.

Roberts SP, Feder ME (1999) Natural hyperthermia and expression of the heat shock protein Hsp70 affect developmental abnormalities in *Drosophila melanogaster*. *Oecologia* 121: 323–329.

Robinet C, Roques A (2010) Direct impacts of recent climate warming in insect populations. *Integr Zool* 5: 132–142.

Rodríguez-Trelles F, Tarrío R, Santos M (2013) Genome-wide evolutionary response to a heat wave in *Drosophila*. *Biol Lett* 9: 20130228.

Sarup P, Frydenberg J, Loeschcke V (2009) Local adaptation of stress related traits in *Drosophila buzzatii* and *Drosophila simulans* in spite of high gene flow. *J Evol Biol* 22: 1111–1122.

Sørensen JG (2010) Application of heat shock protein expression for detecting natural adaptation and exposure to stress in natural populations. *Curr Zool* 56: 703–713.

Sørensen JG, Dahlgaard J, Loeschcke V (2001) Genetic variation in thermal tolerance among natural populations of *Drosophila buzzatii*: down regulation of Hsp70 expression and variation in heat stress resistance traits. *Funct Ecol* 15: 289–296.

Tewksbury JJ, Huey RB, Deutsch CA (2008) Putting the heat on tropical animals. *Science* 320: 1296–1297.

Tweedie S, Ashburner M, Falls K, Leyland P, McQuilton P, Marygold S, Millburn D, Osumi-Sutherland D, Schroeder A et al. (2009) FlyBase: enhancing *Drosophila* Gene Ontology annotations. *Nucleic Acids Res* 37: D555–D559.

United States Fish and Wildlife Service (2013) Endangered species database. http://www.fws.gov/endangered/ (2 September 2013, date last accessed).

Uvarov BP (1931) Insects and climate. *Entomol Soc Lond* 79: 174–186.

Vitousek PM, Loope LL, Stone CP (1987) Introduced species in Hawaii. biological effects and opportunities for ecological research. *Trends Ecol Evol* 2: 224–227.

Woodworth BL, Atkinson CT, LaPointe DA, Hart PJ, Spiegel CS, Tweed EJ, Henneman C, Lebrun J, Denette T, Demots R et al. (2005) Host population persistence in the face of introduced vector-borne diseases: Hawai'i amakihi and avian malaria. *Proc Natl Acad Sci USA* 102: 1531–1536.
